# Research on road parametric modeling and dynamic lightweighting methods driven by BIM-GIS integration

**DOI:** 10.1371/journal.pone.0340062

**Published:** 2026-01-13

**Authors:** Yan Cao, Xinyi Liu, Rui Huang, Menglan Zhu, Zhu Wang, Peng Xu

**Affiliations:** 1 Research Institute of Engineering Safety Science/Reservoir&Dam Center, POWERCHINA GUIYANG Engineering Corporation Limited, Guiyang, China; 2 School of Geography & Environmental Science, Guizhou Normal University, Guiyang, China; 3 Department of Natural Resources of Guizhou, Real Estate Registration Center, Guiyang, China; Henan Polytechnic University, CHINA

## Abstract

Addressing the challenges of inflexibility and low modeling efficiency in the forward design of road building information modeling (BIM), as well as the performance bottlenecks within integrated BIM-GIS environments, this study proposes a novel integration-driven method for parametric modeling and dynamic lightweight processing of roads (PMDL). The proposed approach integrates terrain-adaptive algorithms with lightweight rendering techniques, thereby enabling rapid design iteration and dynamic optimization. Based on this method, a parametric system for 3D road model features is constructed according to the spatial topological relationships between the road and the terrain. Shape grammar is employed to drive the road pavement modeling process, ensuring both design flexibility and modeling efficiency. For complex terrain conditions, a threshold-triggered dynamic modeling mechanism is designed. Through terrain elevation analysis, the undulation of road sections is automatically identified. Algorithms are developed for continuous road pavement generation, Grid-based segmentation of the slopes, dynamic calculation of pier heights, and automatic tunnel generation, enabling the adaptive creation of roads, bridges, tunnels, and slopes. Finally, quadric error metrics (QEM) mesh simplification, level of detail (LOD), and view frustum culling are applied to optimize the loading efficiency and rendering performance of 3D models on the OpenSceneGraphEarth (OSGEarth) platform. This achieves a stable frame rate above 50 FPS for large-scale scenes, effectively resolving rendering lag issues in large-scale scenarios. Experiments show that compared to traditional oblique photography modeling (OPM) and differential elements method (DiEM), this method significantly improves modeling speed, accuracy, and data scheduling efficiency, providing efficient technical support for intelligent design, dynamic updating, and multi-scale visualization of digital twin roads.

## 1 Introduction

With the progression of the economy and the advancement of information technology, China has progressively transitioned into an era characterized by rapid information development, as well as a phase focused on the development and establishment of new foundational surveying and mapping practices. It is against this backdrop that digital twin technology emerges. Digital twin refers to the digitization of elements in the physical world to construct a corresponding virtual world in computer space. It is a dynamic digital technology that reflects the full lifecycle of an entity system, integrating methods such as physical modeling, data transmission, and information sharing [1–4]. As one of the most fundamental and critical infrastructures for pedestrians and various modes of transportation, the research on three- dimensional (3D) models of road entities plays a vital role in engineering planning and construction, urban transportation, natural disaster avoidance, and prediction. It serves as a crucial foundation in the construction of digital twin cities [5–7]. In recent years, the rapid development of building information modeling (BIM) and geographic information system (GIS) technologies has enabled efficient and reliable spatial data integration and 3D geographic information visualization capabilities [8,9]. In the field of 3D road modeling, advancements in BIM-GIS integration technology have made rapid, efficient, and precise parametric 3D modeling of roads possible [10–13]. Traditional road modeling methods rely on manual creation within modeling software, which is labor-intensive, inefficient, and offers limited flexibility and visualization. In the era of digital twins, various modeling methods for geographic entities have been proposed. Current research on 3D geographic entity modeling can be broadly categorized into two approaches: data-driven and model-driven.

Data-driven modeling involves involves detecting the edges, boundaries, and point-line-surface features of the modeling object, analyzing the relationships among points, lines, and surfaces (such as point-to-point, line-to-point, surface-to-point, line-to-line, surface-to-line, and surface-to-surface relationships), and subsequently constructing a complete three-dimensional model through complex processing of the acquired data sources. Of these, the core representative of the data-driven approach is the unmanned aerial vehicle (UAV)-based Oblique Photography Modeling (OPM) method [14,15]. In the 1960s, Kersten T.H. first utilized 3D laser point clouds to study the geometric modeling of buildings [16]. Remondino, from an application perspective, summarized the advantages of UAV systems in providing high spatiotemporal resolution imagery, particularly for large-scale projects and the 3D information acquisition of non-planar objects [17]. Zhang et al. proposed a UAV-based panoramic oblique photogrammetry method. Utilizing a panoramic image projection algorithm, their approach achieved the simultaneous acquisition of georeferenced panoramic images and realistic 3D models [18]. To supplement semantic information in the 3D city reconstruction process, Xu et al. introduced a method for the semantic segmentation of 3D model data derived from oblique photography, enabling the synchronous completion of individual model reconstruction and building outline extraction [19]. Yang et al. integrated UAV oblique photography with LiDAR point clouds to achieve high-precision and lightweight 3D road modeling [20]. Xing et al. utilized UAVs to establish an image dataset of road pavement cracks under complex backgrounds and achieved a significant improvement in detection accuracy through an improved YOLOv5 model [21]. Beyond these applications, the OPM method is also extensively used in various industries such as engineering surveying [22–25], power line inspection [26–29], building extraction [30–32], and smart cities [33–35]. According to existing research, although modeling techniques based on laser point clouds and oblique photography can rapidly acquire surface geometric information, they suffer from lengthy data acquisition and processing cycles, a heavy reliance on the quality of field data sources, and poor flexibility. For instance, point cloud density is highly susceptible to drastic changes in terrain, image-based modeling is easily interfered with by vegetation occlusion, the fine details of models are often lacking, and post-processing steps like individualization are still required. These limitations restrict the further application and in-depth utilization of detailed 3D entity models in areas such as the design and construction of smart engineering projects, the development and management of smart cities, and fundamental GIS services.

The model-driven approach primarily involves constructing a model library and matching the most suitable model structure and graphic element combinations for modeling. Additionally, specific grammars can be used to describe models, and model properties can be altered by adjusting model parameters. Over the years, as the integration of GIS and BIM has deepened, an increasing number of scholars have focused their research on road three-dimensional reconstruction based on the model-driven approach, particularly on parametric road modeling [36,37]. Regarding parametric modeling methods for roads, the primary approach involves extracting the geometric features of model components to form parametric modeling knowledge. These parameters are then parsed to generate the desired geometric models, followed by mapping texture files onto the model surfaces. A classic example is the differential element method (DiEM), which adopts a BIM component assembly concept. Furthermore, BIM models enable digital virtualization of various buildings and equipment through precise geometric structures and rich semantic information. They can fully display model information and support the storage and management of models throughout their entire lifecycle [38,39]. GIS, on the other hand, facilitates the visualization of large-scale scenes and spatial analysis of geographical areas [40]. Unlike BIM, GIS places greater emphasis on showcasing macro-environmental information and spatial models of the external areas surrounding the object of study [41]. The integration of BIM and GIS enables comprehensive design and integrated management of 3D road models, thereby addressing the issues of limited information content in GIS 3D models and the isolation of BIM models, and achieving comprehensive integration of spatio-temporal information [42,43]. Currently, research on the fusion of BIM and GIS primarily revolves around the industry foundation classes (IFC) and city geography markup language (CityGML) data standards [44,45]. Using these standards as carriers, model fusion based on model geometric semantic information and semantic mapping is achieved through data union, mapping, extension, and other methods [46–48].

Supported by BIM and GIS technologies, research on road modeling in recent years has gradually shifted towards generating road models that reflect the real world, with greater emphasis on detailed modeling of road elements [49]. In 2014, Wang et al. converted existing GIS data that only contained 2D road centerline information into high-fidelity 3D road network models, automatically generating basic road elements such as road segments, intersections, and overpasses to form complex road networks [50]. In 2018, Cura et al. developed a framework to produce a coherent street network model, incorporating traffic information and street objects to construct road pavement [51]. Carneiro et al. combined historical and current state data of road infrastructure maintenance to explore the possibilities and potential of GIS, BIM, the internet of things (IoT), and augmented/virtual reality technologies in managing and maintaining road infrastructure [52]. Jorge et al. generated a hybrid BIM-GIS model for Madrid’s Calle 30 through a semi-automated process, bringing the ring road closer to a digital twin of the infrastructure [53]. D’Amico et al. innovatively integrated geometric and design information with road infrastructure monitoring data into a road pavement management system, achieving the preliminary construction of a BIM-based digital twin model [54]. Zhang et al. proposed a comprehensive analysis method based on BIM and GIS, developing an intelligent operation and maintenance platform for highways that effectively addresses data silos in highway maintenance management [55]. While BIM-GIS integrated model-driven road modeling methods offer advantages in model refinement and visual integration, there remains significant room for optimizing 3D road modeling efficiency and rendering performance on this basis.

In summary, although existing methods have made considerable progress in the three-dimensional modeling of road geographic entities, a critical bottleneck persists in the forward design process of road planning and design: an irreconcilable tension between data-driven and model-driven approaches. On the one hand, data-driven methods, represented by OPM, can provide highly realistic 3D models of actual scenes. However, they exhibit significant rigidity when facing design adjustments during the planning phase, and their output models often suffer from data voids and geometric adhesion, making them difficult to use directly for refined design. On the other hand, model-driven methods, exemplified by the DiEM method, offer parametric potential. Yet, their model libraries typically lack adaptability to complex terrain, resulting in geometric errors (e.g., model penetration, fractures) in mountainous areas with significant elevation changes. Furthermore, they generally overlook rendering performance optimization within large-scale GIS environments, failing to meet the demanding requirements of digital twin applications for large-scale, high-frame-rate visualization.

To break through this bottleneck, this study proposes a novel BIM-GIS integration-driven framework—a parametric modeling and dynamic lightweight (PMDL) method for roads. Its distinctive advantage lies in unifying flexibility, accuracy, and performance through the synergy of three key innovations: (1) A semantics-driven parametric model, constructed based on shape grammar, grants the model unprecedented design flexibility and reconstruction efficiency. (2) A terrain-adaptive generation algorithm employs threshold-triggered dynamic modeling algorithms to perceive and respond to terrain fluctuations in real-time, ensuring the seamless and precise integration of road, bridge, tunnel, and slope with the actual terrain. (3) A GIS-oriented dynamic lightweight technology integrates mesh simplification, level of detail (LOD), and view frustum culling, providing the necessary performance guarantee for smooth interaction in large-scale digital twin scenes. The core contribution of this research is the organic integration of these three previously isolated components into an automated workflow, offering an innovative solution that balances flexibility, high accuracy, and high performance to address the central conflict in the forward design of road engineering.

## 2 Materials and methods

### 2.1 Research framework

This paper takes the parametric design of 3D road pavement as the research object. Firstly, based on the spatial relationship between road engineering and terrain, the shape grammar and semantic rules of road pavement are constructed to design the characteristic parameters of the road pavement model. The road pavement model is then generated according to these key characteristic parameters. For sections with undulating terrain, the slope range is automatically acquired and gridded, and the road slope model is generated through mesh computation, 3D construction, and error verification, which, together with the road pavement model, forms a complete road model. In low-lying and mountainous road sections, we design a threshold-triggered dynamic modeling mechanism, terrain-adaptive rule calculations are performed based on designed threshold variations, automatically generating roads, bridges, or tunnels. Finally, to improve the generation, loading, and rendering efficiency of the road model, lightweight operations such as triangular mesh information simplification, LOD hierarchical loading, and view frustum culling are performed on the generated complete road model. This model is then integrated and visualized in the OpenSceneGraphEarth (OSGEarth) engine.

**Fig 1:**
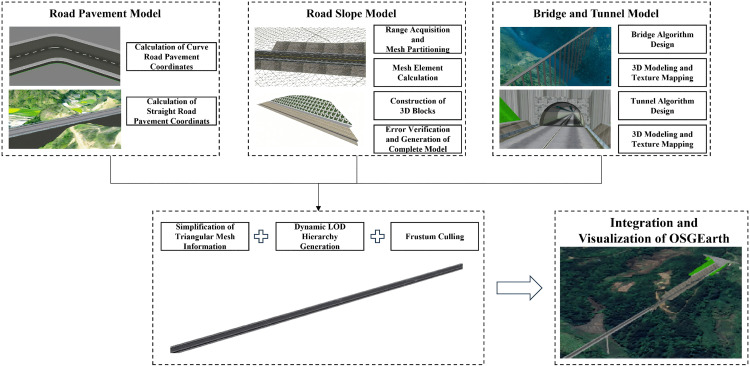
Research framework. The base imagery data consists of UAV orthophotos independently collected by our research team, with a spatial resolution of 0.5 meter and licensed under CC BY 4.0.

Based on the integration of the OSGEarth open-source 3D engine, this study has independently developed a 3D GIS design platform for engineering applications. This platform is capable of efficiently loading and integrating the display of vector, terrain, imagery, oblique, BIM, and other types of data. The algorithmic research and development achievements in this study have all been functionally integrated onto this platform. The research framework of this paper is as follows (Fig 1).

**Fig 2:**
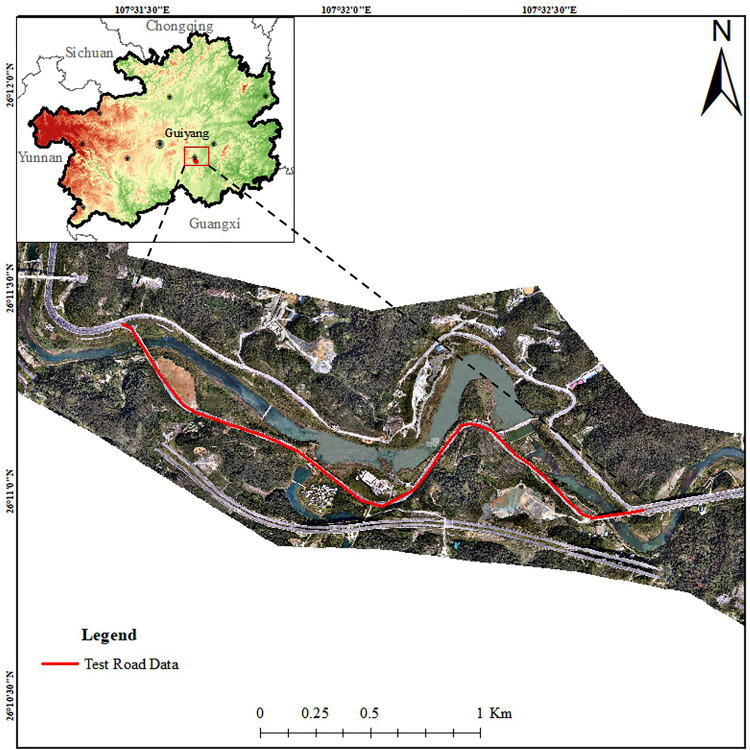
The location map of the verification area. The base imagery data consists of UAV orthophotos independently collected by our research team, with a spatial resolution of 0.5 meter and licensed under CC BY 4.0.

To further validate the effectiveness of the proposed method, a completed 2.8 km-long section of a two-way four-lane road in the mountainous area of Duyun City, Guizhou Province, is selected for testing (located between 107°31′30″E and 107°32′40″E, and between 26°11′0″N and 26°11′30″N), the location map of the verification area is shown below (Fig 2).

### 2.2 Data source and pre-processing

The terrain data for the 3D basemap in the study originates from ASTER GDEM data (https://gdemdl.aster.jspacesystems.or.jp/index_en.html), with a DEM resolution of 30 meters. This data, known as Advanced Spaceborne Thermal Emission and Reflection Radiometer Global Digital Elevation Model (ASTER GDEM), was jointly launched by National Aeronautics and Space Administration (NASA) and Ministry of Economy, Trade, and Industry (METI) of Japan in 2009. It is derived from the detailed observations of the Terra satellite, a new generation of Earth observation satellites by NASA. The sensors used to produce this data include 1.3 million stereoscopic images collected by ASTER. The ASTER mapping data covers all land areas between 83°N and 83°S, making it suitable as terrain basemap data for the rapid parametric design of 3D road pavement in GIS for this study.

The base imagery data introduced in the Results and Discussion sections was independently collected and processed by our research team using UAV orthophotos. The fieldwork was conducted using a DJI Mavic 3E UAV, and the post-processing software used was Smart3D. The data was acquired in August 2021, covering an area of approximately 2.7 km^2^. This dataset is licensed under CC BY 4.0. For access to and use of these data, please contact the corresponding author of this paper.

During the platform development of the integrated engineering 3D GIS design in this study, the VS2015 Integrated Development Environment (IDE) was utilized. The development language employed was C/C++, while the QT framework was used for the development of the interactive interface. The open-source library OSGEarth was adopted for the 3D geographic information system, PostgreSQL was used for database storage, and Redis was utilized for caching data storage. System charts were presented using Echarts, and the graphical underlying layer was implemented based on OpenGL. Notably, the core engine used in this study, OSGEarth, is an extensible terrain rendering toolkit for OpenSceneGraph (OSG). It is an open-source, high-performance, 3D graphics toolkit specifically designed for GIS applications, capable of loading and rendering large-scale 3D terrains and geographical data. The OSGEarth code can be accessed at the following URL: https://github.com/gwaldron/osgearth.

In terms of data preprocessing, due to the large swath width of imagery and terrain data, it is necessary to crop the imagery according to the size of the study area. Additionally, in order to facilitate the integrated loading of imagery, terrain, and other data in this study, it is essential to uniformly define and convert the data coordinate system. This study uniformly adopts the World Geodetic System 1984 (WGS-84) geographic coordinate system. All of the above data preprocessing tasks in this study were completed in QGIS.

### 2.3 Methods

#### 2.3.1 Core concepts and terminology.

Parametric modeling: The parametric modeling method utilizes parametric technology to automatically control and generate 3D models of entities based on their morphological parameters. Parametric technology is a computer-aided design technique that allows flexible control of the geometric features of entities by adjusting parameter values [56,57].

Level of detail (LOD): LOD is used to describe the complexity of geographic object representations. Its purpose is to reduce the geometric complexity of objects during visualization to improve performance. By creating multiple versions of a model with varying levels of detail, the appropriate version is dynamically selected for rendering based on the user’s distance from the model or the viewpoint.

Dynamic lightweight Processing: This refers to dynamically adjusting the level of detail and rendering strategy of a model during runtime based on real-time requirements and environmental changes. By integrating mesh simplification, LOD technology, and view frustum culling, dynamic lightweight processing of BIM models can be achieved. This significantly improves model loading and rendering speed while maintaining visual quality and reducing hardware resource demands.

Digital elevation model (DEM): A DEM is a raster model that digitally represents surface elevation using limited elevation data. Each cell in the grid stores the absolute elevation value of its corresponding location [58].

#### 2.3.2 Design of geometric parameters for algorithms.

In parametric modeling, the design of characteristic parameters is paramount to determining the quality of the final outcome. During the modeling process, parameters are thoroughly decomposed based on topological relationships and object characteristics, yielding detailed modeling design parameters. This parameter design involves the design and constraints of parameters that drive the generation algorithms. Road design primarily encompasses the design of components such as road pavement, bridges, bridge piers, tunnels, and slopes. Depending on their topological structures, these various components can be further broken down into more intricate sub-components. Each component contains common parameters such as texture materials, geometric appearance parameters, starting and ending positions, among others. Detailed road modeling parameters can be found in S1 Fig. Prior to the generation of road components, this paper divides road planning points into segments such as road pavement, slopes, bridges, and tunnels based on topographical and geomorphological characteristics. Each component is then generated according to its corresponding algorithm. This paper developed and integrated algorithms for road pavement generation, slope generation, tunnel generation, and bridge generation.

**(1) Algorithm for road pavement generation**

1) Continuous road pavement generation algorithm

The continuous pavement generation algorithm necessitates consideration of road continuity, particularly in the generation of ramps, turns, and other complex sections. Since the parameters of each planned point are relatively independent, the algorithm must be locally independent. In other words, the road pavement generation should be achievable by solely considering the two adjacent planned points, ensuring that it does not disrupt previous design achievements and minimizes computational time and space costs.

(a)  Generation of curved road pavement coordinates

**Fig 3:**
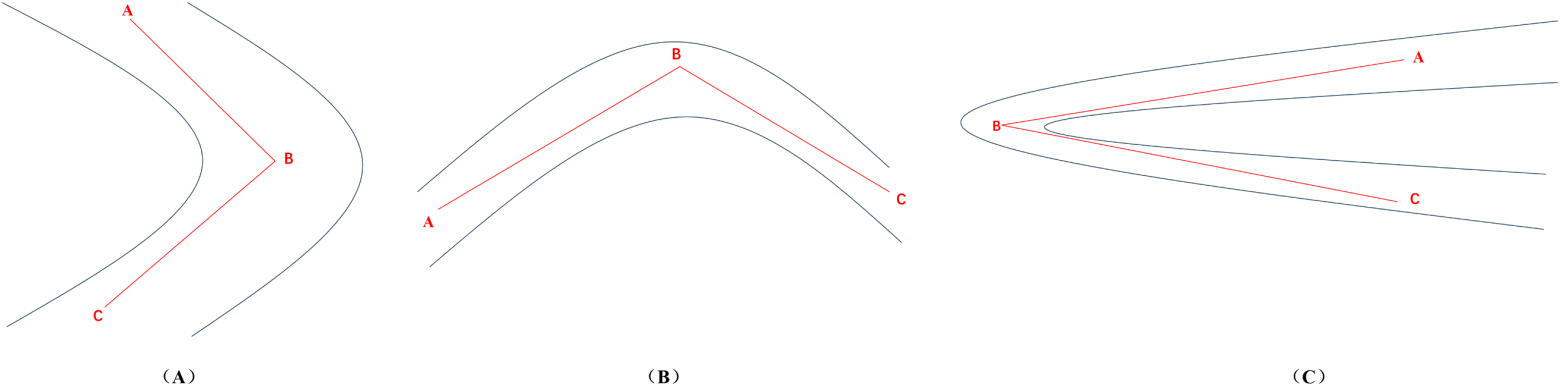
Typical scenarios in curve design. (A) horizontal turns; (B) uphill/downhill turns; (C) folded ramps with height differences.

In 3D scenarios, road design often encounters curves with varying heights, sharp turns, and other complexities. These turns can broadly be categorized into horizontal turns, uphill/downhill turns, and folded ramps with height differences (as shown in Fig 3). This paper proposes a curve interpolation algorithm to accomplish the generation of curved pavement.

When calculating the curve, it requires the assistance of the two points before and after the current point, making a total of three points involved; these points are sequentially labeled as Points A, B, and C; these points are then projected into the local coordinate space with Point B serving as the reference point.

**Fig 4:**
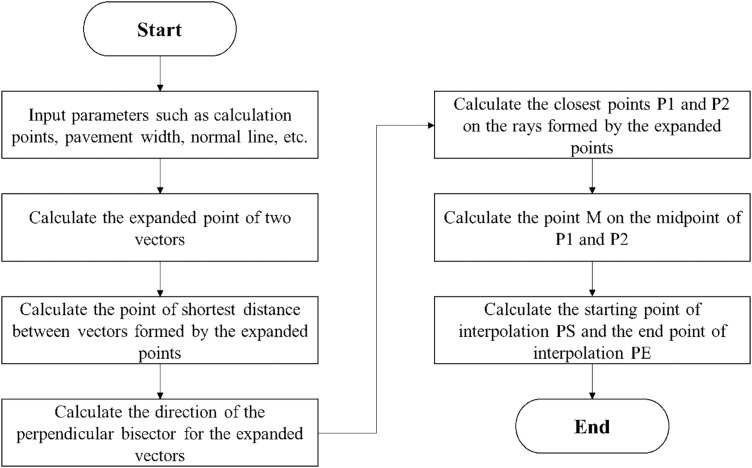
Algorithm process for generating coordinates of curved pavement.

This algorithm is symmetric, meaning the same algorithmic approach is employed on both sides of the road. Taking the algorithm for the left side of the road as an example, the flow of this algorithm is outlined below (Fig 4).

PS and PE denote the interpolation start and end points, respectively. Based on the interpolation resolution parameter, spherical interpolation is performed with point P3 as the center, starting from PS and ending at PE, to calculate the interpolated points along the curve. Starting from point Ax, points are sequentially pushed onto the stack until point Cx is reached, thereby obtaining the complete set of curve coordinates. The same algorithm can be applied to generate the coordinate points on the right side of the curve. Detailed pseudocode and algorithm implementation are provided in S1 Text.

(b)  Generation of 3D road model

Based on the above algorithms, generate all key point data to complete the coordinate generation for the continuous road pavement. Further interpolation and refinement of these coordinate points are necessary for the generation of the road pavement. Due to the symmetry of the algorithm, the number of points on both edges of the road will be the same after calculation, making it easier to complete the interpolation of coordinates based on the interpolation resolution parameter. The calculation of the triangulated irregular network (TIN) and texture coordinates for the road is then performed based on parameters such as road pavement thickness. The detailed process is as follows:

**Step 1**: Based on the interpolation resolution parameter r, calculate the interpolated coordinates for the continuous road pavement along the straight segments on both the left and right sides of segment ABC.
**Step 2**: Based on the road pavement thickness parameter d and the interpolated road pavement coordinates, calculate the triangulated irregular network (TIN) model of the road.
**Step 3**: Calculation of road pavement Texture Coordinates:
  ① The texture coordinates are calculated based on the road sidelines obtained from the above-mentioned curve generation algorithm, denoted as M.
  ② Calculate t1=M/t to obtain the texture coordinates (s, t1), where s is designated as 0 for the left side of the road and 1 for the right side.
**Step 4**: Based on the texture coordinates, the road pavement texture is mapped to each coordinate.
**Step 5**: Based on the length of the road sidelines, obtain the anchor point positions for the guardrail at fixed intervals.
**Step 6:** Based on the guardrail coordinates, generate the guardrail model point by point. The detailed process of road pavement generation is provided in S2 Fig.

After applying the above algorithms and texturing, the basic appearance of the road is formed. Since the road pavement is considered as a single entity, the subsequent generation of models for bridges, tunnels, and other structures will no longer require separate consideration for the corresponding road pavement portions.

2) Slope generation algorithm

The generation of slopes primarily adopts a terrain meshing and partitioning approach. Based on the design parameters for slope generation, the triangular mesh of the slope is calculated according to the algorithmic relationships between different levels and the intersection lines between the slope and the excavated terrain. The slope is then generated by applying texture mapping based on the texture style specified in the slope parameters. By treating the entire algorithm as a function, the inputs are the design parameters and the terrain model, and the output is a 3D slope model. The core algorithmic formula is as follows:


$${Model}_{slope}=F(H_{base},\propto ,W_{i},n,\ldots ,T(x,y),\left\{TextureA\right\})$$ (1)

Here, ${Model}_{slope}$ represents a complete 3D slope model, $H_{base}$ denotes the base elevation of the foundation, ∝ is the slope angle, $W_{i}$ represents the width of each platform level, n indicates the number of slope levels, T(x,y) is the terrain model, and {TextureA} represents the texture style collection. The detailed process of slope generation is provided in S2 Text.

**(2) Bridge algorithm design**

Bridge parameters encompass three major modules: bridge approach parameters, bridge deck parameters, and pier parameters. The semantic information is expressed in detail, encompassing components such as piers, guardrails, and bridge bodies. Customized parameter settings can be applied to each planning point based on the characteristics of the project area, including parameters like pier type, pier texture, pier base, interpolation resolution, guardrail type, and bridge deck type. In the design of bridge algorithms, particular attention must be paid to low-lying areas along the planned route, the length of these areas, bridge approach locations, pier construction, and texture types to ensure the adaptability and flexibility of 3D model generation.The detailed construction process for bridges is outlined as follows:

① Calculation of depressed areas

The road planning route is first mapped out, and the road is subsequently divided into n segments based on the intersections between the planning route and the terrain. The depressed areas, which refer to sections where the terrain height is lower than the planned route, are then identified. Assuming the average height of the i-th segment of the planned route is denoted as h_plan, and the terrain height function is represented as h_terrain(x,y,z (where x, y, z are the coordinates of the terrain), the depressed areas meet the following condition:


low_lying_areas={(x,y,z)|h_terrain(x,y,z)<h_plan(x,y,z)} (2)

Wherein, (x,y,z) represents a point on the terrain, and h_plan(x,y,z) can be obtained through interpolation or retrieved from the planned route data. The above steps are reiterated until all n segments of the road are traversed, and road segments where the formula result is true are designated as Rm and input into the next stage for calculation.

② Determination of the necessity for bridge construction

The traversed road segments from the previous step are further examined. Only when there exists a continuous depressed road segment of a certain length within the planned route, will that segment be designated for bridge construction. Rm(Lrepresents the length of the depressed segment in road segment Rm. If Rm(L)>A (where A is a threshold value), a bridge is required and designated as Ri. Conversely, if Rm(L)<A, bridge construction is not necessary, and the construction of an ordinary road should be considered.

③ Calculation of bridge approach location

For road segments Ri requiring bridge construction, the bridge approach is positioned by extending a certain length B from the intersection point of the bridge and the terrain. The latitude and longitude coordinates of the bridge approach are calculated accordingly. Assuming that the elevation of the bridge approach and the bridge end remains essentially constant, the latitude and longitude of the intersection point between the bridge and the terrain are denoted as (lat_start, lon_start). By simplifying the extension in a certain direction, the latitude and longitude of the bridge approach can be calculated as follows:


lat_end=lat_start+delta_lat*B/distance_per_degree_lat (3)

lon_end=lon_start+delta_lon*B/distance_per_degree_lon (4)

Where delta_lat and delta_lon are the components of the direction vector in latitude and longitude, respectively, and distance_per_degree_lat and distance_per_degree_lon represent the actual distances corresponding to each degree of latitude and longitude, respectively.

④ Pier construction

Firstly, it is necessary to determine whether piers are required for the bridge. Assuming the total length of the bridge is L_bridge, if L_bridge>C (where C is a threshold value), then piers are necessary; otherwise, they are not required. The number of piers, A, can be calculated by dividing the bridge length by the span of a single pier (rounding down or up). When L_bridge>C, the following equation holds:


A=ceil(L_bridge/span_of_pier) (5)

Then, based on the number of piers to be constructed, A, the locations of the piers are calculated. The pier locations can be evenly distributed along the bridge, and the position of the i-th pier, P_i, is calculated as follows:


P_i=start_position+i*(L_bridge/(N+1)) (6)

Here, start_position represents the starting position of the bridge.

Subsequently, the height of each pier is calculated by determining its terrain elevation. The height of the pier, H_pier, is determined by the terrain elevation and the deck height:


H_pier=h_bridge−h_terrain(P_i) (7)

Where h_bridge is the designed height of the bridge deck.

Finally, based on the pier heights and positions, the pier geometries are modeled, the texture mapping coordinates are calculated, and the texture mapping is applied.

⑤ 3D Modeling and texture mapping

Based on the bridge approach locations and the bridge deck width, the geometric modeling of the bridge deck is conducted, and its dimensions are calculated. Simultaneously, texture calculations and mapping are performed. For elevated terrain at the bridge approach, terrain modifications are also required, and slope modeling and texture mapping are conducted using the methods for slope construction described earlier in this paper. The detailed algorithm for texture mapping is described in S3 Text.

**(3) Tunnel algorithm design**

Tunnel parameters primarily encompass three components: portal model, tunnel model, and portal slope parameters. The semantic information is expressed in detail, including portal length, portal width, interpolation accuracy, tunnel radius, texture material parameters, etc. In the algorithm design process, since tunnels are primarily generated when the road is located at a significant height from the mountain surface, the generation of tunnels requires calculations from the points of entry and exit from the mountain. By utilizing the tunnel radius, all coordinate points for the tunnel’s generation are computed, and a 3D tunnel is created by applying textures. At the entrance and exit of the tunnel, excavations need to be made in the terrain to ensure the tunnel’s connectivity.

The detailed construction process of the tunnel is as follows:

① Calculation of highland areas

The roadway is divided into n segments based on the intersection points between the planned route and the terrain, allowing for the identification of low-lying areas, where the terrain height is below the planned route. Similarly, the highland areas, where the terrain height exceeds the planned route, are calculated using the intersection points. Assuming the average height of the k-th segment of the planned route is denoted as h_plan(x,y,z) (where (x,y,z) are the coordinates of the terrain), and the terrain height function is hterrain (x,y,z), the highland areas satisfy the following condition:


high_ground_areas={(x,y,z)|h_terrain(x,y,z)>h_plan(x,y,z)} (8)

(x,y,z)represents points on the terrain, and the road segments for which the formula result is true are designated as Rt and fed into the next stage for further calculation.

② Determination of tunnel construction necessity

The road segments passed through in the previous step are further iterated. Only when a continuous highland segment of a certain length exists within the planned route, will that segment be considered for tunnel construction. Rt(L) denotes the length of the highland segment within the road segment Rt. If Rt(L)>B (where B is a threshold value), then a tunnel construction is required, designated as Rk. Conversely, if Rt(L)<B, the segment is considered for construction as an ordinary road.

③ Calculation of tunnel portal locations

For road segments Rk that require tunnel construction, based on the starting intersection point between the tunnel and the terrain, let the intersection be denoted as (x_intersect, y_intersect, z_intersect). The tunnel portal is determined as the point where the terrain height exceeds the threshold B above the planned route height. Assuming the tunnel entrance and exit elevations for this segment are approximately the same, B represents an increment relative to the planned route height at that point, with its geographical coordinates being (x_tunnel_entrance, y_tunnel_entrance, z_tunnel_entrance). Thus, the following condition holds true when:


x_tunnel_entrance=x_intersect (9)

y_tunnel_entrance=y_intersect (10)

z_tunnel_entrance=z_intersect (11)

Otherwise, the current road segment is designed as an ordinary road with slopes, and the algorithm automatically proceeds to the next road segment for calculation. The algorithm for entering and exiting the tunnel portal is consistent.

④ Calculation of tunnel model starting point

A fixed length C is extended outward from the tunnel portal location to determine the starting point of the tunnel model. Assuming that the elevation of the tunnel model’s starting point remains unchanged from that of the tunnel portal, let the coordinates of the model’s starting point be (x_start, y_start, z_start). Then:


x_start=x_tunnel_entrance−C*cos(direction_angle) (12)

y_start=y_tunnel_entrance−C*sin(direction_angle) (13)

z_start=z_tunnel_entrance (14)

Where direction_angle is the angle between the direction of tunnel extension and a reference direction (e.g., due north).

⑤ Tunnel portal modeling, texture mapping, and terrain clipping

The extended portions of the tunnel entrances and exits are constructed similar to ordinary roads. To ensure the safe operation of both the ordinary roads and the tunnel, it is necessary to construct slopes and modify the terrain. The slope levels on both sides are calculated separately. Following the slope construction methods outlined in previous sections, slope modeling and texture mapping are performed. This ensures that the transitions between the tunnel portals and the surrounding terrain are visually coherent and meet safety requirements. The detailed algorithm for texture mapping is described in S3 Text.Utilizing the locations of the tunnel entrances and exits, as well as the tunnel radius R, a semi-cylindrical tunnel is modeled. The starting and ending points of the tunnel body coincide with the positions of the entrance and exit portals, respectively, with a radius of R. Based on the locations, widths, and thicknesses of the tunnel portals, terrain clipping is performed to excavate the area occupied by the tunnel from the surrounding terrain. Finally, texture mapping is applied to the modeled tunnel to generate a visually realistic representation.

#### 2.3.3 Model lightweighting processing.

To enhance the loading and rendering efficiency of BIM models in GIS platforms, model lightweighting is essential. This process reduces model information complexity, compresses data dimensions, and increases the level of abstraction, while preserving model accuracy and functional completeness.

**(1) Triangular mesh information simplification**

The geometric information of the model is stored in a triangular mesh, where complex BIM models often require millions of triangular meshes to depict model features. Such a vast amount of data poses significant challenges for the transmission and rendering of the model on the Web. Therefore, this paper employs the Garland edge collapse algorithm based on QEM to simplify the triangular mesh [59,60].

The edge collapse algorithm initially computes the cost of folding each edge in the model, sorts these costs in ascending order, and iteratively removes and folds the edge with the lowest cost from the front of the queue. After folding an edge, all associated edges are removed from the priority queue, their folding costs are recalculated, and edges that meet the folding criteria are reinserted into the queue in priority order. This process continues until no edges meet the folding criteria. The Garland algorithm, specifically, is an edge collapse algorithm based on the quadric error metric, which utilizes the sum of squares of distances from a vertex to its associated triangular planes as the error measure. This algorithm is known for its speed, high quality of simplification, and overall efficiency as an edge collapse method. The detailed implementation process is outlined below.

The error Δ(v) for folding a vertex $v={\left[v_{x}v_{y}v_{z}\text{1}\right]}^{T}$ to a new vertex $\overset{―}{v}$ is defined as:


$$\Delta (v)=\sum {\mathrm{\backslash nolimits}}_{P_{v}}{\left(P^{T}v\right)}^{\text{2}}=\sum {\mathrm{\backslash nolimits}}_{P_{v}}v^{T}\left(PP^{T}\right)v=v^{T}\left(\sum {\mathrm{\backslash nolimits}}_{P_{v}}K_{p}\right)v$$ (15)

Where: $p=[abcd{]}^{T}$, with ax+by+cz+d=0, and $a^{\text{2}}+b^{\text{2}}+c^{\text{2}}=\text{1}$;

$P_{v}$represents the set of planes associated with v; $K_{p}$ denotes the fundamental error of p which is:


$$K_{p}=PP^{T}=[\begin{array}{cc} \begin{array}{cc} a^{\text{2}} & ab\\ ab & b^{\text{2}} \end{array} & \begin{array}{cc} ac & ad\\ bc & bd \end{array}\\ \begin{array}{cc} ac & bc\\ ad & bd \end{array} & \begin{array}{cc} c^{\text{2}} & cd\\ cd & d^{\text{2}} \end{array} \end{array}]$$ (16)

We define $Q_{v}$ as the quadratic error measurement matrix for vertex v, as shown below:


$$Q_{v}=\sum {\mathrm{\backslash nolimits}}_{P_{v}}K_{p}$$ (17)

During the initialization process, since each initial vertex is the intersection point of its associated triangles, the initial error Δ(v) for the original vertex v is 0. When performing an edge collapse$\text{O}\left(v_{ep\text{1}},v_{ep\text{2}}\right)\to \overset{―}{v}$, the edge collapse cost and optimal position of the new vertex v are given as follows:


$$\Delta \left(\overset{―}{v}\right)={\overset{―}{v}}^{T}\left(Q_{ep\text{1}}+Q_{ep\text{2}}\right)\overset{―}{v}$$ (18)

$$\overset{―}{v}=[\begin{array}{cc} \begin{array}{cc} q_{\text{1}\text{1}} & q_{\text{1}\text{2}}\\ q_{\text{2}\text{1}} & q_{\text{2}\text{2}} \end{array} & \begin{array}{cc} q_{\text{1}\text{3}} & q_{\text{1}\text{4}}\\ q_{\text{2}\text{3}} & q_{\text{2}\text{4}} \end{array}\\ \begin{array}{cc} q_{\text{3}\text{1}} & q_{\text{3}\text{2}}\\ \text{0} & \text{0} \end{array} & \begin{array}{cc} q_{\text{3}\text{3}} & q_{\text{3}\text{4}}\\ \text{0} & \text{1} \end{array} \end{array}]-\text{1}\left[\begin{array}{c} \text{0}\\ \text{0}\\ \text{0}\\ \text{1} \end{array}\right]$$ (19)

Where: $Q_{ep\text{1}}$ and $Q_{ep\text{2}}$ are the quadratic error measurement matrices for $v_{ep\text{1}}$ and $v_{ep\text{2}}$ respectively. After the collapse, the quadratic error measurement matrix $Q_{\overset{―}{v}}$ for the new vertex v is:


$$Q_{\overset{―}{v}}=Q_{ep\text{1}}+Q_{ep\text{2}}=[\begin{array}{cc} \begin{array}{cc} q_{\text{1}\text{1}} & q_{\text{1}\text{2}}\\ q_{\text{2}\text{1}} & q_{\text{2}\text{2}} \end{array} & \begin{array}{cc} q_{\text{1}\text{3}} & q_{\text{1}\text{4}}\\ q_{\text{2}\text{3}} & q_{\text{2}\text{4}} \end{array}\\ \begin{array}{cc} q_{\text{3}\text{1}} & q_{\text{3}\text{2}}\\ q_{\text{4}\text{1}} & q_{\text{4}\text{2}} \end{array} & \begin{array}{cc} q_{\text{3}\text{3}} & q_{\text{3}\text{4}}\\ q_{\text{4}\text{3}} & q_{\text{4}\text{4}} \end{array} \end{array}]$$ (20)

**Fig 5:**
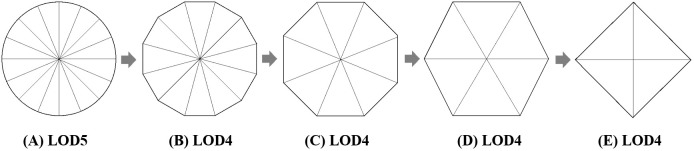
Simplification of triangular mesh information.

The edge collapse algorithm simplifies the model grid evenly, allowing for adjustable LOD to achieve varying model resolutions (Fig 5).

**(2) Dynamic LOD hierarchy generation**

The original road BIM generated experiences severe lag and slow rendering speeds during browsing in 3D engines. Due to the limited capacity of the engines, spatial partitioning of BIM models with complex components and large data volumes is necessary.

Based on the fundamental process of the Garland edge collapse algorithm, which performs well in terms of model simplification effects, there is still room for improvement in efficiency. Therefore, this paper leverages the characteristics of the edge collapse algorithm to partition the railway model into multiple regions and then applies edge collapse operations to each region individually.

**Fig 6:**
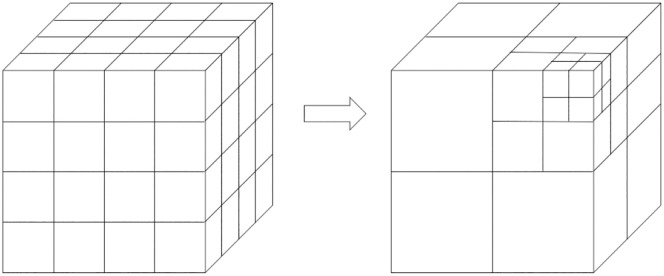
Comparison between uniform grid division and octree grid division.

Model space partitioning generally involves two methods: uniform and non-uniform grid partitioning, with octree partitioning belonging to the latter category [61,62]. The figure below compares uniform grid partitioning with octree-based non-uniform grid partitioning (Fig 6). Uniform grid partitioning divides the overall bounding box of the model into sixty-four smaller bounding boxes of equal size, then further divides the first-level bounding boxes into eight equally-sized grids, and so on, as illustrated in the diagram.

The specific steps are as follows:

1) Calculate the root node coordinates of the model’s maximum bounding volume (where m represents the current model). The formulas for the model’s length W, width H, and height D are as follows.

$$W=m_{max}X-m_{min}X$$ (21)

$$H=m_{max}X-m_{min}X$$ (22)

$$D=m_{max}X-m_{min}X$$ (23)

Taking the largest-sized scene as the root node, its coordinates are::


$$\left(m_{min}X+\frac{W}{\text{2}.\text{0}f},m_{min}Y+\frac{H}{\text{2}.\text{0}f},m_{min}X+\frac{D}{\text{2}.\text{0}f}\right)$$ (24)

**Fig 7:**
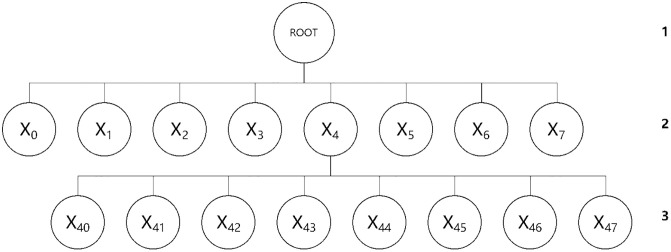
Illustration of octree node division rules.

2) Extend from the root node in the XYZ directions of space to divide the maximum bounding volume into eight equal-sized regions, which are referred to as child nodes. Place the face elements from the object file format (OBJ) model into the grids without child nodes, and categorize the center point coordinates of the face bounding boxes into the bounding boxes of their respective child nodes.
3) Set the maximum error metric value, the child node face threshold, or the maximum recursion depth based on the number of triangular faces in the model. If the current level is less than the set threshold or has not reached the maximum recursion depth, proceed with the next level of node division. Stop dividing when the number of elements assigned to a child node is not zero and is the same as the number of elements in its parent node. The rules for octree node division are illustrated in the figure below (Fig 7).

**(3) View frustum culling technique**

**Fig 8:**
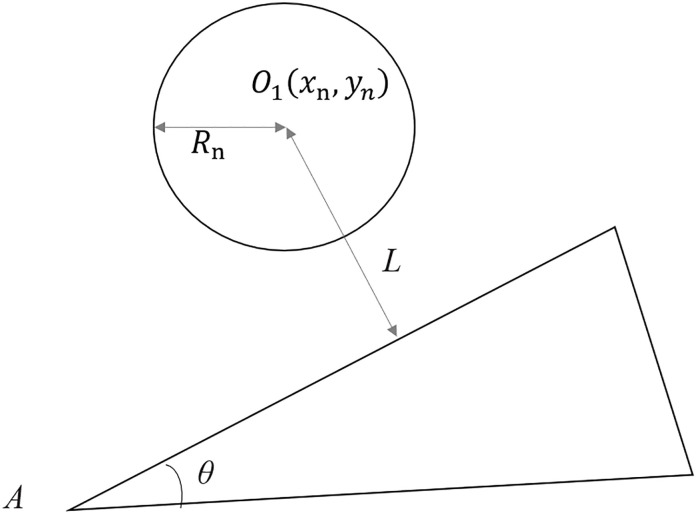
Illustration of frustum culling.


The BIM model of roads generated features massive data and encompasses numerous 3D models, resulting in significant storage space occupation and substantially affecting the efficiency of model rendering. When browsing through 3D models, users can only observe the portions within the field of view displayed on the Web client, and whether the remaining data are loaded has no impact on the final display outcome. Therefore, on top of LOD, introducing the view frustum culling technique can effectively reduce the amount of data loaded for the model and enhance rendering efficiency. Among these, the key components of view frustum culling are frustum culling and screen-space error algorithms.By utilizing an adaptive octree indexing structure, rapid loading of different LOD models across various hierarchical structures can be achieved. On this foundation, the frustum culling algorithm is incorporated to manage changes in camera viewpoints and angles. This algorithm determines whether a node’s 3D tile should be included in the rendering sequence based on its position and geometric error, thereby further enhancing the efficiency of scene rendering [61,62]. The design principles of this algorithm are outlined below. In the following figure (Fig 8), $O_{\text{1}}$ represents the center of the tile bounding box, $(x_{n},y_{n}\backslash )$are the coordinates of point $O_{\text{1}}$, $R_{n}$ is the approximate radius of the bounding box, A denotes the viewpoint, θ is the angle of the view frustum, and L is the shortest distance between the frustum boundary and point $O_{\text{1}}$, which can be derived from the following formula:

$$L=\left(y_{n}-x_{n}tan\frac{\theta }{\text{2}}\right)cot\frac{\theta }{\text{2}}$$ (25)

**Fig 9:**
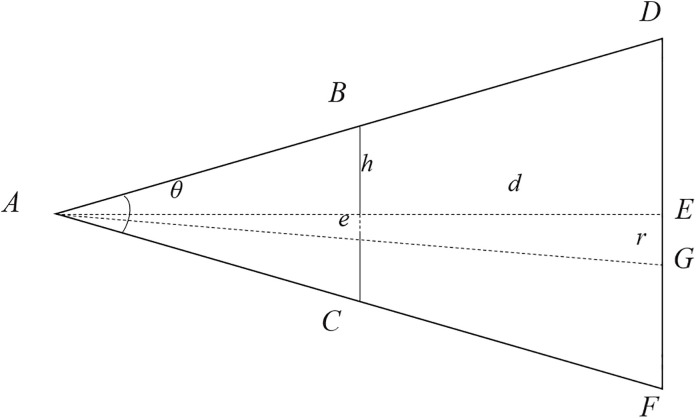
Illustration of screen-space error.

The value of L is compared with $R_{n}$. If $L<R_{n}$, the tile is loaded; otherwise, the tile is not loaded.For models within the view frustum, the resolution of the 3D tiles to be loaded is determined based on their distance from the viewpoint. The closer a model is to the viewpoint, the higher the resolution (i.e., LOD level) of the tiles required; conversely, the farther a model is from the viewpoint, the lower the resolution (i.e., LOD level) of the tiles required. During runtime, the screen-pace error is calculated based on the geometric error of the tiles, as illustrated in the figure below (Fig 9).

In the figure above, the distance r between E and G represents the predefined spatial geometric error of the tile, d is the distance from the viewpoint to the far clipping plane AE, h is the screen height, and e is the screen-pace error. The geometric relationship between e and r is as follows:


$$\frac{e}{r}=\frac{h}{\text{2}d\cdot tan\frac{\theta }{\text{2}}}$$ (26)

Using the above formula, the screen-pace error e can be calculated. By comparing e with a preset threshold α, if e > α, the next level of tiles is loaded to replace the current tiles; otherwise, the current tiles are retained.

In terms of road model management, CityGML is an international standard for extensible urban spatial data exchange and encoding, published by ISO TC211 and OGC. It follows object-oriented principles and supports hierarchical descriptions of the same geographic entity, as well as integrated storage and management of geometric and semantic features. Although the road entity models in this study possess numerous parametric features, they exhibit a stable structure and a clear relationship with terrain. Therefore, in organizing and managing road models, a hierarchical parametric approach is adopted in accordance with the CityGML standard [62].

## 3 Results and disscussion

### 3.1 Results

**Fig 10:**
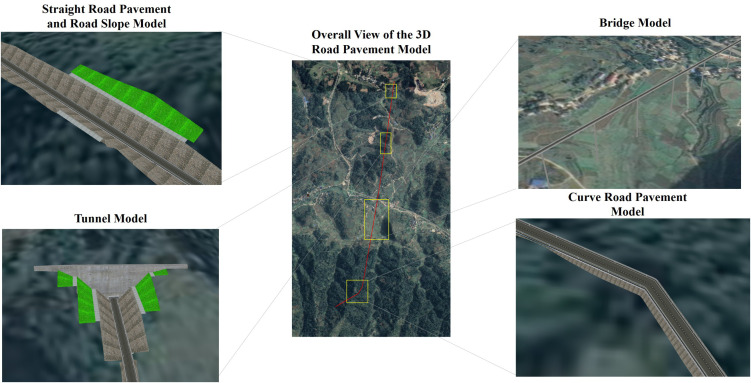
Results of parametric design for 3D road pavement. The base imagery data consists of UAV orthophotos independently collected by our research team, with a spatial resolution of 0.5 meter and licensed under CC BY 4.0.

This study conducted functional integration and visualization implementation on an engineering 3D GIS design platform built using the OSGEarth engine. Incorporating the algorithms developed in this study, the platform exhibits significant advantages in rapidly deploying road layouts and comparing schemes during the engineering planning and design phase. Specifically, by inputting measured terrain data from different project areas and integrating them uniformly within the 3D scene, the platform can achieve seamless integration of various engineering structures, such as ordinary roads, slopes, bridges, and tunnels, with the terrain under different topographical conditions. The modeling results for roads, slopes, bridges, and tunnels are illustrated in the figures below (Fig 10).

**Fig 11:**
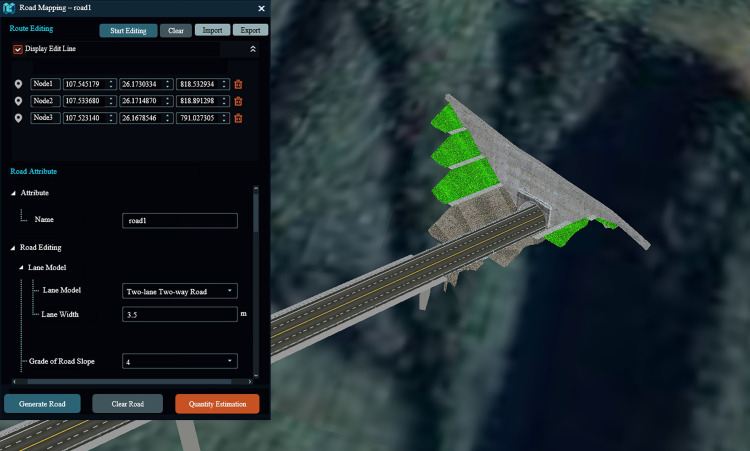
Route editing parameter settings. The base imagery data consists of UAV orthophotos independently collected by our research team, with a spatial resolution of 0.5 meter and licensed under CC BY 4.0.

**Fig 12:**
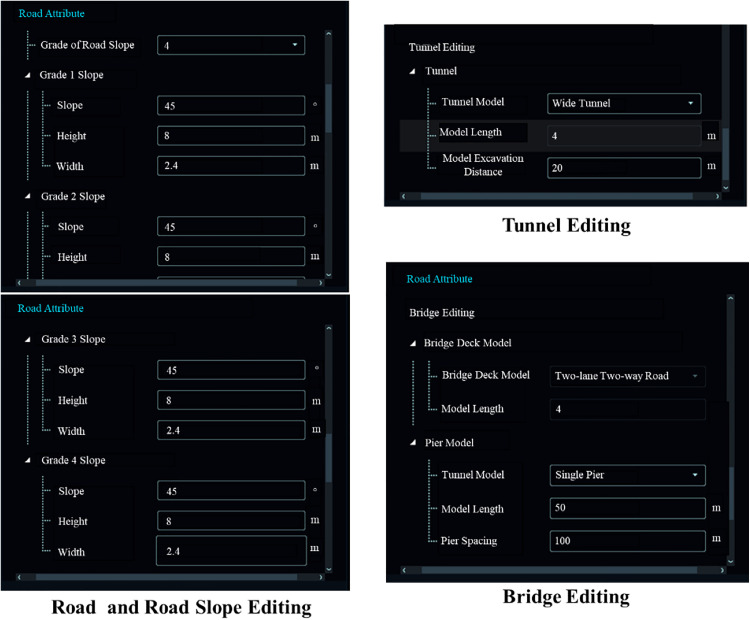
Road attribute parameter settings.

The GIS-based 3D road pavement rapid parametric design platform offers a high degree of flexibility by enabling customized modification of modeling parameters and rapid generation of models. The figure below illustrates the parameter input during the 3D road pavement modeling process, which is divided into the route editing and road attribute modules (Figs 11, 12). In route editing, users can either input road planning path files in vector formats (such as.shp,.kml, etc.) through importation, or draw road paths in real-time by clicking on the 3D base map overlaid with terrain and imagery. Nodes can be drawn to adjust the path in real-time, and the set path can be cleared or exported as a vector file.

The Road Attribute Module further comprises three parameter setting options: Road and Slope Editing, Bridge Editing, and Tunnel Editing. In Road and Slope Editing, users can configure lane models, such as two-lane two-way, four-lane two-way, six-lane two-way, etc., and customize the width of the lane model. Additionally, the slope design level in road editing is a crucial parameter in the 3D road pavement modeling process. By pre-setting the slope levels, including customizing the slope, height, and width of slopes from level 1–4, precise integration of slopes with roads and terrain during 3D road pavement model construction can be achieved.In the bridge editing module, the lane type parameter of the bridge deck model default to those of the road lane model, ensuring consistency. The pier model allows for the selection of single or double piers, with customizable lengths and spacings. In the tunnel editing module, users can choose between wide and narrow tunnel models, with the model length matching that of the road. The excavation distance parameter specifies the threshold height above which tunnel construction is required, and can be customized.Finally, for the constructed 3D road pavement, the platform utilizes terrain-based cut-and-fill algorithms to provide a preliminary estimation of road construction quantities.

The GIS-based rapid parametric design platform for 3D road pavement efficiently integrates multi-source data, achieving a high level of accuracy while delivering realistic and rich visualization effects. Leveraging the OSGEarth open-source engine, this platform integrates various geospatial data sources, including UAV orthophotos, ASTER GDEM 30m default DEM, and self-produced and loadable oblique photography models. This provides an abundant base data source for 3D road modeling, enabling the generated models to more accurately reflect actual scenarios. Furthermore, under the unified WGS-84 coordinate system, high-precision georeferencing is provided for basemap imagery, DEM, oblique photography models, BIM models, and more, ensuring data consistency throughout the model generation, modification, and output processes. Parametric modeling ensures the precision of models in terms of geometry, spatial position, and other aspects, providing a solid foundation for 3D road modeling. Moreover, the generated 3D road models, while achieving precise integration with the terrain environment, simplify model mesh information. Supported by LOD dynamic partitioning and view frustum culling techniques, the platform enables multi-angle display, full-range zooming, 2D-3D switching, and other realistic and rich visualization effects for 3D models.

### 3.2 Disscussion

To validate the effectiveness of the proposed method, a comparative analysis was conducted between the proposed PMDL method and the OPM and DiEM methods under identical testing conditions.The testing hardware environment was standardized on a workstation equipped with an Intel(R) Xeon(R) w3-2435 CPU, 128GB of RAM, and an NVIDIA RTX A4000 GPU. In terms of validation dimensions, the assessment focused on three primary aspects: model construction speed, model construction accuracy, and model loading and rendering performance.

**(1) Model construction speed**

Choosing an appropriate construction method for a 3D road pavement model can significantly impact both the speed and accuracy of model construction. Among the traditional forward design methods for road 3D models, OPM is a data-driven approach that generates high-precision 3D reality models through multi-angle oblique photography. The core principle involves collecting surface imagery from various angles, including vertical and oblique views, and combining these with computer vision algorithms to reconstruct the real-world 3D geometry and texture information.

In traditional forward design approaches for 3D road models, while data-driven methods utilizing oblique photography models or LiDAR technology are prevalent for 3D modeling, model-driven methods based on graphic primitive combinations can also effectively model 3D road pavement. The BIM-GIS integration-driven parametric modeling and dynamic lightweighting method for roads proposed in this paper is one of the model-driven methods. Based on this, this paper reproduces a classic and rapid modeling method within the model-driven approach, known as the DiEM. This method employs the BIM component assembly concept, utilizing a simple road model library to match the planned and designed route. It rapidly combines different parameters to model roads, slopes (or embankments), tunnels, and bridges separately, and finally assembles them to complete the road scene.

**Fig 13:**
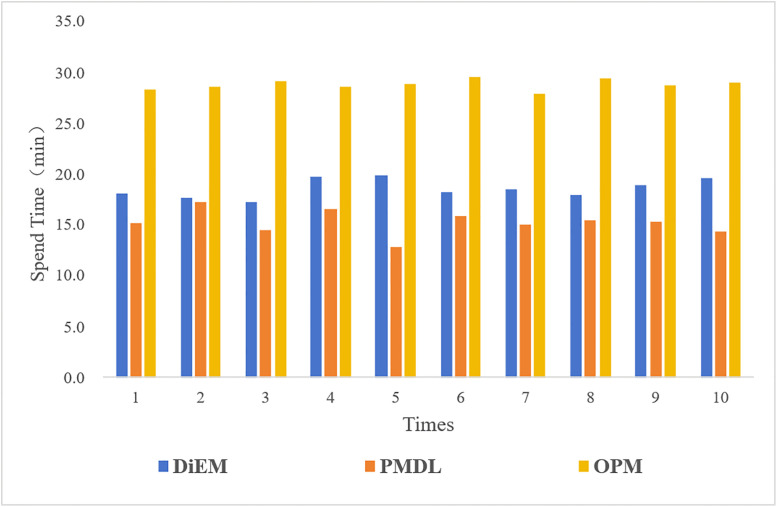
Comparison of construction speeds across different modeling methods.

For comparative validation, the DiEM, OPM, and the PMDL used in this paper were compared. Ten modeling tests were conducted on the same route for each method to compare their modeling speeds. The comparison chart of the construction speeds for different methods is shown below (Fig 13).

The analysis and test results indicate that in the OSGEarth platform, the OPM method requires prior aerial acquisition of the study area, followed by setting multiple parameters such as coordinates and aerial triangulation for the collected data before generating the 3D model (in this study, Smart3D was used for oblique photography model data production). Although this method can generate a highly realistic original 3D scene, due to its large data processing volume and complex construction process, it still consumes the longest time among the methods tested, with an average duration of 28.78 minutes for 10 model constructions. The DiEM method, while capable of directly applying a model component library for 3D construction, encounters numerous conflicts and overlaps when dealing with complex road conditions such as uphill and downhill sections, and sharp turns. To achieve better generation results, the DiEM method needs to continuously splice and model components, resulting in a significant decline in the efficiency and quality of 3D model generation. The average construction speed of DiEM ranks second among the methods, with an average duration of 18.54 minutes for 10 model constructions. Finally, the PMDL method accurately captures the geometric structure and semantic information of the model. Based on the relationship between the model and terrain, it directly generates a 3D road pavement model through real-time calculation and optimization using a semantic rule library and terrain-adaptive generation algorithm, rapidly achieving dynamic model updates and semantic enhancement. This avoids multiple data conversions during data flow and effectively improves model construction efficiency. The PMDL method has the fastest average construction speed among the methods tested, with an average duration of 15.23 minutes for 10 model constructions. Compared to OPM and DiEM, PMDL exhibits an improvement in model generation efficiency by 88.97% and 21.72%, respectively. The raw data from the comparative process are provided in S1 Fig.

**(2) Model construction accuracy and visualization**

**Fig 14:**
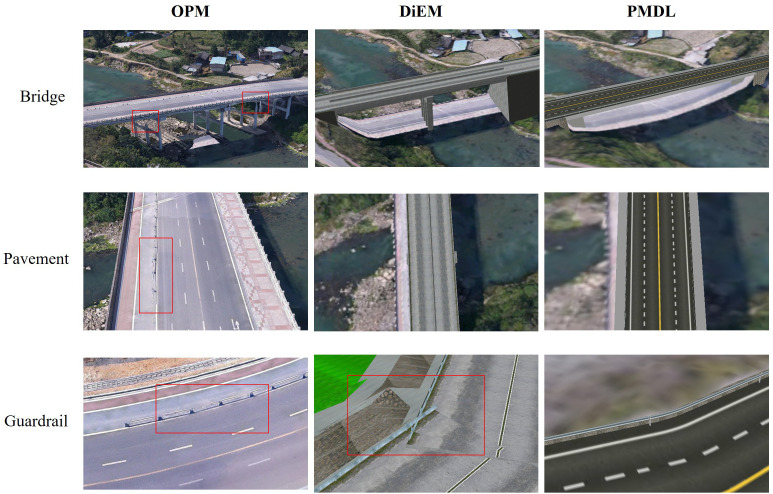
Comparison of accuracy across different modeling methods. The base imagery data consists of UAV orthophotos independently collected by our research team, with a spatial resolution of 0.5 meter and licensed under CC BY 4.0.

The 3D road models constructed for the same road segment using PMDL, DiEM, and OPM are presented as follows (Fig 14).

As observed in the figures, OPM, being based on the acquisition and production of real-world scenes, can provide a realistic representation of completed road entities. However, model accuracy and effectiveness are significantly influenced by factors such as acquisition route, pilot operation, and weather conditions. Due to the acquisition perspective, areas such as under bridges, guardrails, and road details may suffer from missing photographs, leading to ineffective acquisition of ground feature information during aerial triangulation. This often results in issues such as model adhesion and blurred details. Furthermore, oblique photography models can only capture and model existing roads and cannot directly support 3D route selection design during the road planning phase.

DiEM, which employs a method of combining and assembling model components, demonstrates good model performance on straight road sections and aligns well with the environmental terrain. However, on curved and sloping road sections, due to the lack of accurate structural and semantic information, excessive component assembly calculations can lead to errors such as model overlap, penetration, and fragmentation. Additionally, the relatively rough model components result in significant deficiencies in road markings, guardrail details, and an inability to accurately simulate actual road conditions.

In contrast, the PMDL method not only enables custom settings for road paths but also supports adaptive construction of bridges, slopes, or tunnels for planned routes along depressions and highlands. By invoking a semantic rule library to accurately calculate road parameters for turns, slopes, bridges and road pavement details, PMDL has high application value in terms of both model construction accuracy and visualization effects for 3D design route selection in the road engineering planning and design phase. The raw data from the comparative process are provided in S2 Fig.

**(3) Comparison of different data scheduling methods**

**Fig 15:**
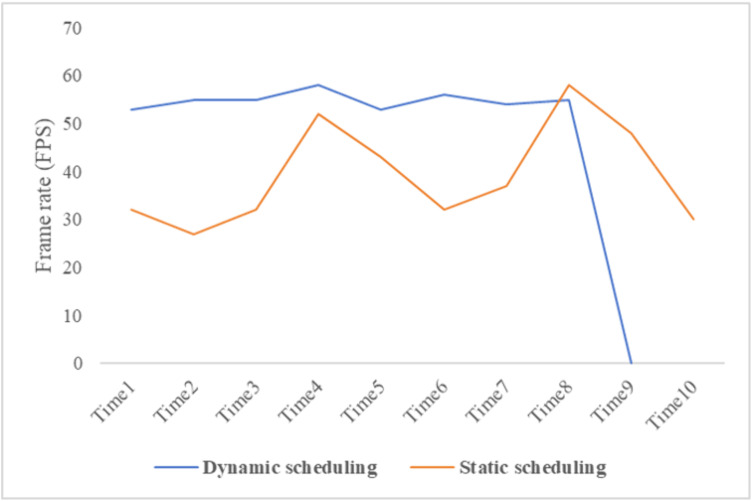
Comparison of different data scheduling methods.

The comparison results of different data scheduling methods are shown below (Fig 15).

After generating the 3D road pavement model, dynamic scheduling of 3D data is achieved through mesh information simplification, octree structures, and view frustum culling technique. This paper compares the performance of dynamically scheduled data in terms of loading and rendering with that of statically scheduled data, as illustrated in the figure above. The figure demonstrates that the dynamic scheduling method maintains a stable frame rate around 50 frames per second (FPS), while the static scheduling method experiences fluctuations in frame rate, hovering around 40 fps.

Throughout the entire data loading process, dynamic scheduling demonstrates greater stability and shorter loading times, with a stable increase in frame rate by approximately 25%. Without implementing octree structures and frustum culling algorithms, the instability in frame rate and the prolongation of loading time would be even more pronounced, particularly when dealing with large amounts of data and under inefficient experimental platforms. Consequently, the adoption of mesh information simplification, octree structures, and view frustum culling techniques can significantly enhance the efficiency of data loading and rendering.

## 4 Conclusions

Addressing the issues of poor flexibility and reusability in the forward planning and design process of current road engineering BIM models, as well as inefficient and labor-intensive 3D BIM model reconstruction, this study proposed a BIM–GIS integration-driven PMDL method for roads. A parametric system for 3D road model features was established based on the spatial relationship between road engineering and terrain. Algorithms for road and slope generation were developed to produce continuous road pavement and slope models according to key characteristic parameters. Depending on various terrain undulation conditions, calculation rules were set to automatically generate BIM models for slopes, bridges, and tunnels within GIS 3D scenarios. Finally, the generated models were lightweighted through mesh simplification, hierarchical LOD loading, and view frustum culling, enabling rapid generation and loading of road BIM models in GIS 3D environments. Compared with existing road 3D construction methods, the proposed method demonstrated significant advantages in modeling speed, accuracy, and loading efficiency within GIS 3D scenarios. The main achievements of this study are as follows:

(1) Based on the spatial relationship between road engineering and terrain, a semantic parameter system was established encompassing basic components, curves, guardrails, slopes, bridges, and tunnels. Constructed on a grammar-based parametric framework, the system provided unprecedented design flexibility and reconstruction efficiency. Through modular decomposition and recombination of parameters, dynamic adaptation of model components was achieved.
(2) A terrain-adaptive algorithm was developed using a threshold-triggered dynamic modeling mechanism to automatically generate road pavement, slopes, bridges, and tunnels during road generation in response to real-time terrain variations, enabling rapid modeling in complex terrain conditions.. While maintaining accurate geometric detail, the proposed method improved model generation efficiency by 88.97% compared to OPM and by 21.72% compared to the DiEM.
(3) To address model data discrepancies resulting from the differing emphasis of BIM and GIS technologies, mesh simplification and LOD algorithms were employed. By integrating view frustum culling, dynamic scheduling and rendering optimization of 3D scene models were achieved. The incorporation of dynamic lightweight processing techniques significantly enhanced model loading efficiency and rendering performance in large-scale digital twin scenarios. Comparative validation showed that the proposed method achieved a 25% higher stable frame rate compared to static scheduling approaches.
(4) Efficient integration of GIS 3D scenarios and road BIM models was achieved on the OSGEarth engine through multi-source data fusion, 3D visualization and rendering, and parametric design technologies, significantly improving road planning and design efficiency as well as visualization quality. This framework is expected to further promote the application of GIS–BIM integrated technology in road engineering, enhance the scientific rigor and efficiency of planning and design processes, and support broader adoption of digital engineering practices.

## 5 Limitations and future work

This study still has some limitations that need to be addressed in the future:

(1) Although this study utilized 30-meter publicly available terrain data for parametric modeling research and validated the method’s effectiveness on a 2.8-km mountainous road section with complex terrain, limitations exist regarding terrain variability. In actual road engineering planning and design processes, project areas exhibit diverse topographic conditions. To more comprehensively evaluate the robustness of the proposed method, future work should test it under more varied terrain conditions, such as plain urban areas with complex intersections and rolling hilly regions. This constitutes an important direction for our future research.
(2) The Garland algorithm takes the quadratic measure of geometric error as the core optimization objective. Although it is efficient in the process of model mesh simplification, it has limitations such as semantic unawareness and insufficient curvature sensitivity. In the next step of research, we will attempt to further optimize the algorithm in this study through methods such as semantic-aware edge folding and neural network assistance.
(3) The texture mapping method in this study enables rapid and efficient texture mapping during the road model generation process. Although it can improve the generation efficiency of road models to a certain extent, texture distortion or visual discontinuity may still occur in irregular road segments with severe terrain fluctuations or during rapid dynamic LOD switching. Due to current algorithmic limitations, more research on texture mapping distortion issues is needed in the future.
(4) This study mainly focuses on the algorithm design of key components such as road pavement, slopes, bridges, and tunnels based on GIS methods during the model construction process. In later research, attention should also be paid to the completeness and richness of detailed components such as guardrails, streetlights, and roadside trees in the road construction process, to enhance the realism and detail restoration capabilities of the model.

## Supporting information

S1 Text The process of curve generation. (DOCX)

S2 Text The process of slope generation. (DOCX)

S3 Text The process of texture mapping. (DOCX)

S1 Fig Detailed parameters information for road pavement modeling. (DOCX)

S2 Fig Schematic diagram of road pavement generation process. (DOCX)
