# Supplementary material for: Research on road parametric modeling and dynamic lightweighting methods driven by BIM-GIS integration
Source: PLoS One. 2026 Jan 13;21(1):e0340062. doi: 10.1371/journal.pone.0340062 (PMC12798999; doi:10.1371/journal.pone.0340062)
Supplement: S1 Text — (DOCX) [file pone.0340062.s001.docx]

**Table A. Schematic of Curve Pseudocode Generation**

Function: Curve Interpolation Algorithm Implementation

**Input**: Three consecutive planning points A, B, C; road width W; normal vector N
**Output**: Interpolation start point PS, interpolation end point PE
**BEGIN**
1: Vab ← B - A; /* Compute vector between A and B */
2: Vbc ← C - B; /* Compute vector between B and C */
3: V0 ← normalize(cross_product(Vab, N)) * W; /* Calculate perpendicular vector to Vab with length W */
4: Ax ← A + V0; /* Compute extended point from A in V0 direction */
5: ABx ← B + V0; /* Compute extended point from B in V0 direction */
6: V1 ← normalize(cross_product(Vbc, N)) * W; /* Calculate perpendicular vector to Vbc with length W */
7: BCx ← B + V1; /* Compute extended point from B in V1 direction */
8: Cx ← C + V1; /* Compute extended point from C in V1 direction */
9: AABx ← ABx - Ax; /* Compute vector between ABx and Ax */
10: BCxC ← Cx - BCx; /* Compute vector between Cx and BCx */
11: ABCM ← mirror(AABx, BCxC); /* Calculate perpendicular bisector of the two extended vectors */
12: R1 ← make_ray(Ax, ABx); /* Construct ray R1 from Ax and ABx */
13: R2 ← make_ray(BCx, Cx); /* Construct ray R2 from BCx and Cx */
14: P1 ← min_distance(R1, R2); /* Find point on R1 closest to R2 */
15: P2 ← min_distance(R2, R1); /* Find point on R2 closest to R1 */
16: M12 ← midpoint(P1, P2); /* Calculate midpoint between P1 and P2 */
17: P3 ← M12 + normalize(ABCM) * W; /* Offset M12 along ABCM direction by distance W */
18: PS ← min_distance(R1, P3); /* Find point on R1 closest to P3 → PS */
19: PE ← min_distance(R2, P3); /* Find point on R2 closest to P3 → PE */
**END**
**Return**: PS, PE

**Step 1**: Input three road design points sequentially labeled as A, B, and C. Input parameters include: road pavement width w, normal vector N, interpolation resolution r (density of curve points), road pavement thickness d, and texture width t.

**Step 2**: Generation of curve road pavement coordinates. When calculating curves, points are projected onto a local coordinate space with point B as the reference point. This algorithm is symmetric, and the same algorithm is used to generate both sides of the road. Taking the algorithm for the left side of the road as an example:

① Connect the key road points A, B, and C to obtain vectors $\vec{AB}$、$\vec{BC}$.

② Calculate the perpendicular vectors of $\vec{AB}$ and $\vec{BC}$ with respect to the normal vector $\vec{N}$, with a length equal to the road pavement width w, to obtain the expanded parallel lines of the road.

③ Calculate the corresponding expansion points Ax, ABx, Cx, and BCx of $\vec{AB}$ and $\vec{BC}$ on the expanded parallel lines of the road.

④ Calculate the vector $\vec{\mathrm{AABx}}$ corresponding to points ABx and Ax, and the vector $\vec{\mathrm{BCxC}}$ corresponding to points Cx and BCx.

⑤ Calculate the perpendicular bisector of $\vec{\mathrm{AABx}}$ and $\vec{\mathrm{BCxC}}$, with a length equal to the road width w, to determine the center point OL for spherical interpolation.

⑥ Construct ray R1 from points Ax and ABx, and construct ray R2 from points BCx and Cx.

⑦ Calculate the points PS and PE on rays R1 and R2 that are closest to the sphere's center point OL, respectively.

⑧ Based on the interpolation resolution parameter r, perform spherical linear interpolation (slerp) with OL as the center, PS as the start point, and PE as the end point. This allows for the calculation of interpolation points along the curve. Starting from the point Ax, these points are sequentially pushed into a stack until the point Cx is also included, thereby obtaining the entire set of coordinates for the left-hand side of the curve.

**Step 3**: Using the algorithm described above, calculate the coordinates for the right side of the curve.
