# Supplementary material for: Research on road parametric modeling and dynamic lightweighting methods driven by BIM-GIS integration
Source: PLoS One. 2026 Jan 13;21(1):e0340062. doi: 10.1371/journal.pone.0340062 (PMC12798999; doi:10.1371/journal.pone.0340062)
Supplement: S2 Text — (DOCX) [file pone.0340062.s002.docx]

The slope generation process is as follows:

(a)Acquiring the calculation scope for slope generation

The process of acquiring the calculation scope for slope modeling primarily encompasses three steps: Firstly, a basic cross-section of the slope is constructed based on slope design parameters such as the base elevation of the slope foundation, slope angle, number of slope levels, platform widths at each level, and drainage ditch width. The starting elevation of the polyline for constructing the basic cross-section is set as the base elevation of the slope foundation, while the ending elevation is slightly greater than the maximum elevation of the mountain. Secondly, using the basic cross-section line, the process is advanced longitudinally along the slope face at a fixed step size, calculating the intersection coordinates between the cross-section line and the terrain surface, as illustrated in the figure below. The (x, y, z) coordinates of each intersection point are recorded, forming a set of intersection point data. Finally, based on the spatial positional relationships between the intersection points, all non-self-intersecting points in the intersection point data set are connected sequentially in either a clockwise or counterclockwise order to form a topologically correct 3D vector surface. This vector surface represents the calculation scope for slope generation.


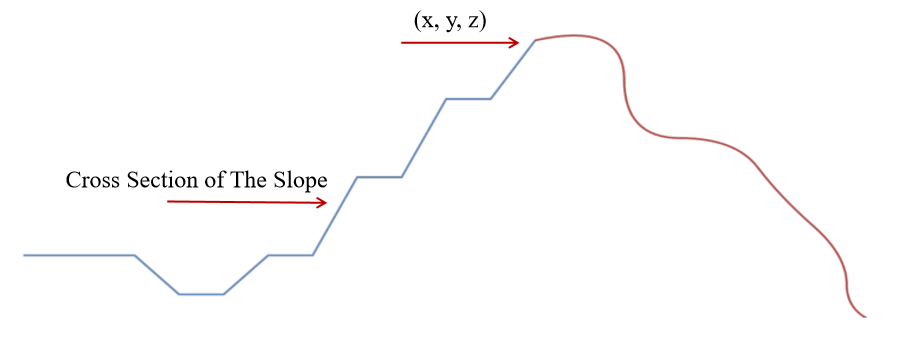


**Figure A. Schematic diagram of intersection points on terrain surface**

(b)Grid-based segmentation of the slope calculation scope

The slope calculation scope is refined and segmented into multiple differential element using fixed-size square grids. Irregular polygons adjacent to boundary lines are expanded into squares, as shown in the figure below. During the calculation of each differential unit, the 3D spatial coordinates of the four vertices of each unit are recorded and stored in a spatial coordinate data set. Subsequently, based on the coordinates of each differential element, all elements are plotted within the 3D geospatial scene, and the results are validated by overlaying the boundary lines of the slope calculation scope. If optimization or further refinement of the segmentation elements is required, the grid dimensions can be adjusted, and the differential calculations are repeated element the level of refinement is satisfactory.


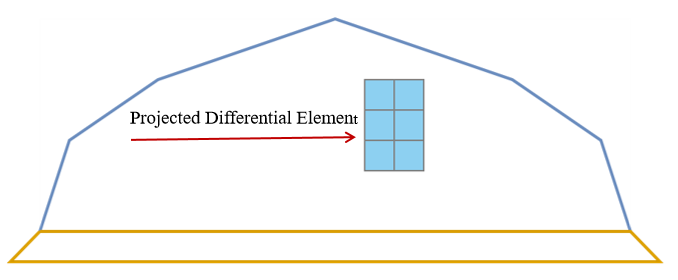


**Figure B. Position of differential element on the slope**

(c)Conducting unit-by-unit calculations based on grid segmentation

Along the longitudinal direction of the slope, at a fixed distance (e.g., 1 meter), cross-section lines are sequentially intersected with the boundary lines of the slope calculation scope to obtain intersection points. The cross-section lines are truncated at these intersection points, resulting in a set of cross-section lines in 3D space that collectively form an irregular slope surface. Based on the results of grid segmentation, the corner point coordinates of each unit are calculated to project the unit onto the irregular slope surface. The projection position then determines the attributes of the differential unit (e.g., drainage ditch, retaining wall, slope, slope platform, etc.).

(d)Construction of 3D volume elements

For differential units with the same attributes, based on their spatial adjacency relationships, adjacent units are progressively calculated and merged. This process results in the formation of a vector surface for differential element sharing the same attribute, as illustrated in the figure below.


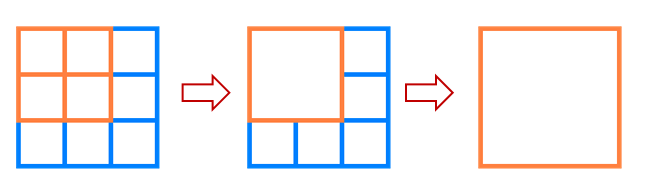


**Figure C. Schematic diagram of differential element merging**

After all differential units with identical attributes have been merged, several vector surfaces with distinct attributes are formed (e.g., drainage ditch surface, retaining wall, first-level slope surface, first-level slope platform surface, second-level slope surface, second-level slope platform surface, etc.). By projecting these vector surfaces onto the cross-sections of the slope, projected surfaces are obtained. A 3D volumetric element in the shape of a hexahedron is then constructed, comprising the spatial vector surfaces, projection surfaces, and four projection side faces, as illustrated in the figure below.


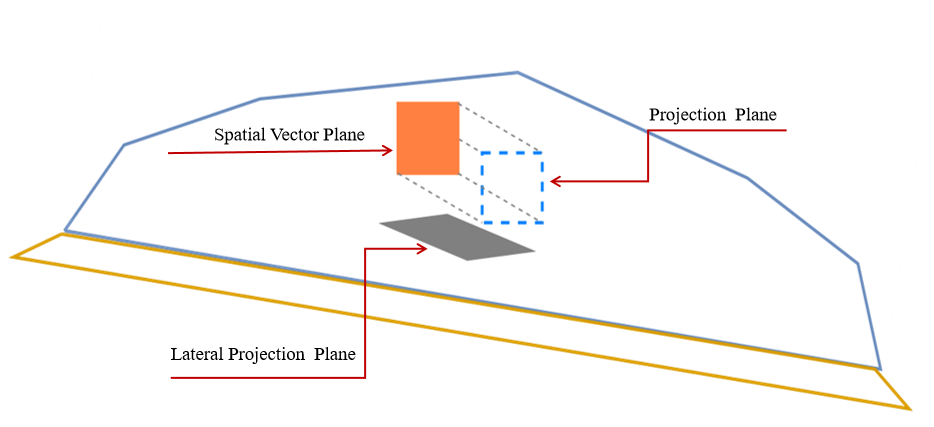


**Figure D.** **Schematic diagram of 3D volumetric element composition**

(e)Constructing a complete slope model

Starting from the top of the slope and proceeding downwards, the distance error between adjacent faces of the 3D volumetric elements is validated. A threshold of 0.1 meters can be set, and if the distance between adjacent faces is less than 0.1 meters, they are considered as validated and merged. After merging, the common faces are removed, resulting in a new 3D volumetric element. This process is repeated until a complete 3D slope model is formed. Textures are then applied to surfaces with mapping texture information (e.g., textures for drainage ditches, retaining walls, first-level slopes, first-level slope platforms, second-level slopes, and second-level slope platforms). Ultimately, a complete 3D slope model with surface textures is obtained, as illustrated in the figure below.


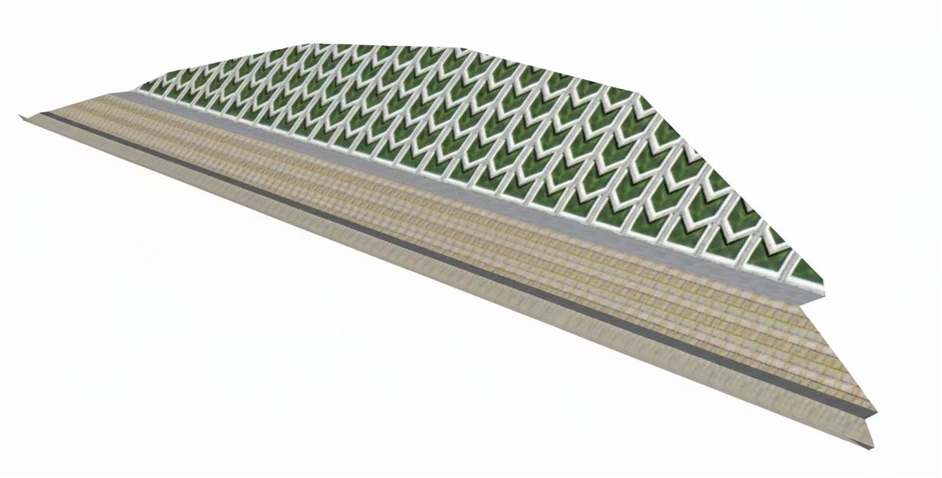


**Figure E. 3D model of the completed merged slope**
