# Supplementary material for: Research on road parametric modeling and dynamic lightweighting methods driven by BIM-GIS integration
Source: PLoS One. 2026 Jan 13;21(1):e0340062. doi: 10.1371/journal.pone.0340062 (PMC12798999; doi:10.1371/journal.pone.0340062)
Supplement: S3 Text — (DOCX) [file pone.0340062.s003.docx]

The texture mapping process is as follows:

The purpose of texture mapping is to render 3D models more visually realistic. Essentially, it involves projecting a two-dimensional texture image onto the surface of a 3D solid model through mapping relationships, as illustrated in the figure below. The texture mapping process based on authentic images of photographed geographical entities is depicted in the subsequent figure. This method enables the precise positioning of texture pixels onto the geometric surface of the model. However, issues such as texture occlusion and uneven color distribution may arise, which detract from the realism of the 3D model.


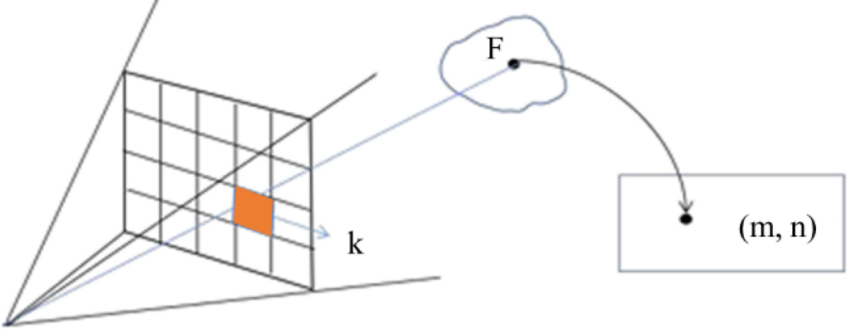


**Figure A. Illustration of texture mapping**

$S\left( k \right)=F$ (1)

$T\left( F \right)=(m,n)$ (2)

In this context, $T$ represents the mapping function from scene space to texture space, while $S$ denotes the mapping function from screen space to scene space. This paper employs pre-processed public textures for texture mapping onto road entity models. For unit road models, the surface is relatively regular, making texture mapping straightforward. However, for road primitives models featuring turns or intersections, applying the texture mapping method designed for regular roads can result in texture distortion. To address this issue, the current study employs an automatic texture stitching and mapping approach. The schematic diagram is shown below.





**Figure B. Texture unit coordinates and texture target area coordinates**

There are three scenarios for mapping texture units onto the surface of road entity models: proportional mapping, repetitive texture mapping, and texture deformation mapping. The texture mapping processes for these three scenarios are as follows:

1) The target region ABCD maintains a proportional relationship with the texture region A_1_B_1_C_1_D_1_, and the target region is textured through a one-to-one mapping correspondence with the texture region.

2) When the target region AEFD is larger than the texture region A_1_B_1_C_1_D_1_, multiple texture mappings are applied to the target region. In the case illustrated above, the ABCD region is first mapped. Subsequently, points B and C serve as new starting points. By calculating the ratio k of BE to AB, the texture is cropped to the texture unit at k, and then texture mapping is performed through vertex matching. For scenarios requiring three or more texture mappings, simply repeat the mapping process for the ABCD region, crop the texture to the texture unit at k, and proceed with texture mapping.

3) When the target region EGHF does not conform to a regular shape identical to the texture shape, such as in the case of road curves or 3D pier surfaces, texture deformation is necessary to align it with the shape of the target region, thereby obtaining texture coordinates for each point. Furthermore, the target region is partitioned into blocks, and all blocks are traversed. Texture mapping is then performed through vertex correspondence across these blocks.
