# Supplementary figures and images for: Research on road parametric modeling and dynamic lightweighting methods driven by BIM-GIS integration
Source: PLoS One. 2026 Jan 13;21(1):e0340062. doi: 10.1371/journal.pone.0340062 (PMC12798999; doi:10.1371/journal.pone.0340062)

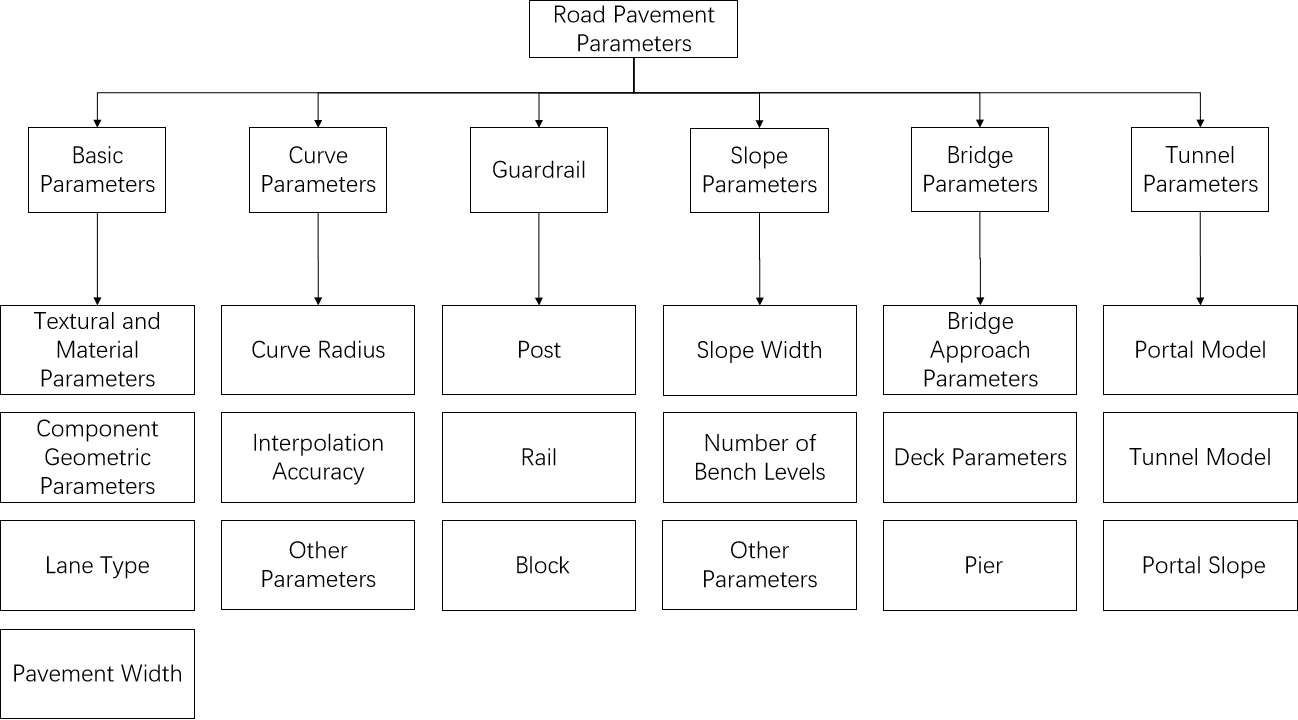


**Figure A. Detailed parameters information for road pavement modeling**

Supplement: S1 Fig — (DOCX) [file pone.0340062.s004.docx]
