# Supplementary material for: Research on road parametric modeling and dynamic lightweighting methods driven by BIM-GIS integration
Source: PLoS One. 2026 Jan 13;21(1):e0340062. doi: 10.1371/journal.pone.0340062 (PMC12798999; doi:10.1371/journal.pone.0340062)
Supplement: S2 Fig — (DOCX) [file pone.0340062.s005.docx]

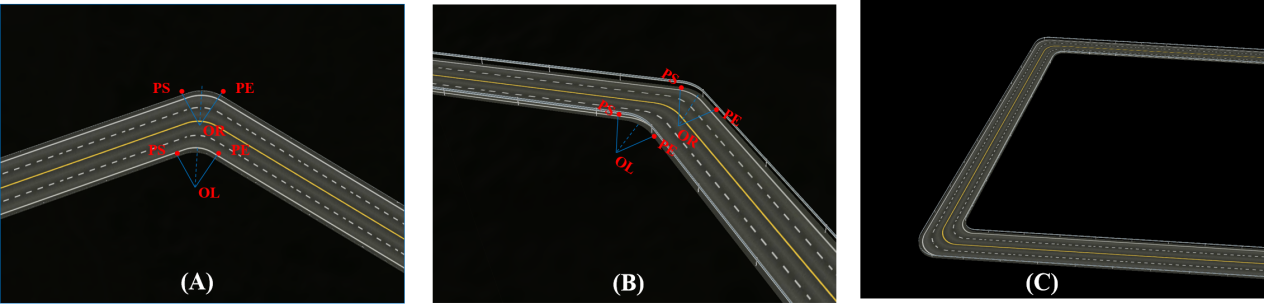


**Figure B. Schematic diagram of road pavement generation process. (A) Interpolation start point (PS) and end point (PE), and left/right curve centers (OL, OR) in horizontal alignment; (B) Interpolation start point (PS) and end point (PE), and left/right curve centers (OL, OR) in vertical alignment; (C) Resulting continuous road pavement.**
